# Integrative PANoptosis gene profiling reveals prognostic and therapeutic insights in prostate cancer

**DOI:** 10.17305/bb.2025.11998

**Published:** 2025-06-19

**Authors:** Yi Wang, Yiheng Du, Xizhi Wang, Jiang Yu, Qing Gu, Guangquan Yuan, Yifei Zhu, Liyang Gu, Jun Ouyang

**Affiliations:** 1 Department of Urology, The First Affiliated Hospital of Soochow University, Suzhou, China; 2 Department of Urology, Suzhou Kowloon Hospital, Shanghai Jiao Tong University School of Medicine, Suzhou, China

**Keywords:** Gene risk signature, prostate cancer, PCa, programmed cell death

## Abstract

Prostate cancer (PCa) remains a significant global health challenge, representing the most common solid tumor in men and the fifth leading cause of cancer-related death. Despite therapeutic advances, achieving a definitive cure remains difficult. Early diagnosis and personalized treatment strategies are crucial for improving patient outcomes. Programmed cell death—particularly PANoptosis, an inflammatory pathway that integrates pyroptosis, apoptosis, and necroptosis—has emerged as a promising therapeutic target in oncology.In this study, individuals with PCa were categorized into PANoptosis-high and PANoptosis-low subgroups based on the expression levels of 45 PANoptosis-related genes (PRGs). Differential gene expression analysis and subsequent enrichment analyses were conducted to explore the biological pathways associated with each subgroup. A four-gene risk signature (CASP7, ADAR, DNM1L, and NAIP) was identified, showing strong predictive value for overall survival (OS) in both training and validation cohorts. This signature was independently associated with OS and showed meaningful correlations with the tumor microenvironment, particularly immune cell infiltration and immunotherapy responsiveness. These findings suggest that the PRG signature may serve as a valuable prognostic biomarker and inform immunotherapeutic strategies in PCa management.

## Introduction

Prostate cancer (PCa) is recognized as the most prevalent solid tumor among men and stands as the fifth leading contributor to cancer-related mortality on a global scale, representing a major public health concern. Genetic predisposition and environmental exposures influence the likelihood of developing PCa, with factors such as advanced age and familial history serving pivotal roles [1–3]. Patients frequently exhibit nonspecific clinical manifestations, including reduced urine flow, urgency, increased nocturia, and a sensation of incomplete bladder emptying, which often result in delayed diagnosis and elevated mortality rates [4, 5]. Although substantial progress has been made in radiotherapy, targeted therapies, and immunotherapy, attaining a definitive cure continues to present significant challenges. The early identification of individuals at elevated risk of recurrence, coupled with prompt therapeutic intervention, can extend survival and enhance quality of life. Thus, developing reliable prognostic biomarkers to facilitate precision medicine, particularly in guiding personalized chemotherapy and immunotherapeutic strategies, remains imperative.

Programmed cell death, encompassing pyroptosis, apoptosis, and necroptosis, is fundamental to preserving homeostasis and influencing disease progression. The emergence of PANoptosis, an inflammatory programmed cell death pathway orchestrated by the PANoptosome complex, is distinguished by its integration of pyroptotic, apoptotic, and/or necroptotic features [6–10]. This phenomenon has been associated with a spectrum of pathological conditions and has garnered substantial attention in cancer research. Evidence indicates that PANoptosis plays a role in multiple cancer-related biological processes, including tumorigenesis and resistance to chemotherapy in colorectal cancer, as well as modulating the response to immunotherapy in gastric cancer [11–16]. Prior studies have demonstrated that the combination of TNF-α and IFN-γ exhibits efficacy in targeting various tumor cell types, underscoring its potential clinical applications. The cooperative interaction between TNF-α and IFN-γ has been shown to activate multiple signal transduction pathways, including *GSDMD, GSDME,* caspase-8, and *MLKL*. The activation of PANoptosis in tumor cells via this mechanism has been recognized as a pivotal process in suppressing tumor initiation and restricting tumor progression, presenting opportunities for targeted therapeutic interventions [17–19]. Nevertheless, PANoptosis does not exclusively exert anti-tumor effects; in certain contexts, it may contribute to tumor progression. For instance, elevated caspase-8 levels within the nuclei of tumor cells have been reported to inhibit intrinsic apoptotic pathways while simultaneously promoting mitotic activity, thereby facilitating tumor development. Conversely, caspase-8 has also been identified as a downstream effector of granzyme, particularly facilitating GSDME cleavage and triggering pyroptosis, which enhances anti-tumor immune responses and impedes tumor proliferation [20]. Thus, further investigation into the function of PANoptosis in tumor biology holds substantial promise for clinical advancements.

This study is designed to elucidate the fundamental molecular mechanisms underlying PANoptosis-related genes (PRGs) in PCa. A systematic analysis of their expression patterns will be conducted using data from The Cancer Genome Atlas (TCGA) and Gene Expression Omnibus (GEO) datasets to identify PRGs that correlate with PCa prognosis. Through the development of prognostic models, insights into the tumor microenvironment (TME) status will be explored, along with predictions regarding patient responsiveness to immunotherapy. The findings are expected to assist in discovering novel prognostic biomarkers and treatment targets for PCa.

## Methods and materials

### Data sources

The datasets and samples related to PCa utilized in this study were acquired from multiple publicly available repositories. For the training cohort, RNA-seq data from 544 PCa patients were retrieved from the TCGA database (accessible at https://portal.gdc.cancer.gov/). The raw read counts were converted into transcripts per kilobase million (TPM) values. Furthermore, DNA methylation profiles and genetic mutation data were obtained from cBioPortal. Information on clinicopathological characteristics and overall survival (OS) was accessed via the UCSC Xena browser (https://xenabrowser.net/datapages/).

An external validation set was constructed using the gene expression profiling dataset GSE70770, which comprises 203 PCa samples along with their corresponding clinical data. This dataset was acquired from the GEO database (accession number: GSE70770; https://www.ncbi.nlm.nih.gov/geo/query/acc.cgi?acc=GSE70770) [21, 22]. The microarray data obtained from GSE70770 were log2-normalized.

### Collection of PRGs and development of protein–protein interaction (PPI) network

The profiles of PRGs (Table S1) were obtained from previously published studies [23, 24]. To investigate the interactions among PRGs, the STRING database (https://cn.string-db.org/) was employed for PPI network analysis [25].

### Consensus clustering

To classify molecular subtypes associated with PRGs, unsupervised consensus clustering was conducted using the “ConsensusClusterPlus” package in R [26]. This analysis was based on the expression profiles of these genes in PCa specimens. To determine the most appropriate number of clusters, various cluster sizes ranging from *k* ═ 2 to *k* ═ 10 were evaluated, with the consensus clustering procedure repeated 1000 times to enhance result stability and accuracy. The optimal clustering parameter was identified through systematic evaluation of the cumulative distribution function (CDF) curve, the consensus matrix, and a consistency score exceeding 0.9. Additionally, the “pheatmap” function in R was used to visualize the clustering results in heatmap format.

**Table 1:** Gene-specific forward and reverse primer sequences (5′–3′)

**Gene**	**Forward primer sequence (5′–3′)**	**Reverse primer sequence (5′–3′)**
*CASP7*	CGGAACAGACAAAGATGCCGAG	AGGCGGCATTTGTATGGTCCTC
*ADAR*	TCCGTCTCCTGTCCAAAGAAGG	TTCTTGCTGGGAGCACTCACAC
*DNM1L*	GATGCCATAGTTGAAGTGGTGAC	CCACAAGCATCAGCAAAGTCTGG
*NAIP*	CCGAACAGGAACTGCTTCTCAC	CCACAGACAGTTCTTTCAGGCAC
*CDH1*	GCCTCCTGAAAAGAGAGTGGAAG	TGGCAGTGTCTCTCCAAATCCG
*CDH2*	CCTCCAGAGTTTACTGCCATGAC	GTAGGATCTCCGCCACTGATTC
*VIM*	AGGCAAAGCAGGAGTCCACTGA	ATCTGGCGTTCCAGGGACTCAT
*BAX*	TCAGGATGCGTCCACCAAGAAG	TGTGTCCACGGCGGCAATCATC
*CASP1*	GCTGAGGTTGACATCACAGGCA	TGCTGTCAGAGGTCTTGTGCTC
*GSDMD*	ATGAGGTGCCTCCACAACTTCC	CCAGTTCCTTGGAGATGGTCTC
*GAPDH*	GTCTCCTCTGACTTCAACAGCG	ACCACCCTGTTGCTGTAGCCAA

Abbreviations: *CASP7*: Caspase 7; *ADAR*: Adenosine deaminase acting on RNA; *DNM1L*: Dynamin 1 like; *CDH1*: Cadherin 1; *CDH2*: Cadherin 2; *VIM*: Vimentin; *BAX*: Bcl-2 associated X protein; *CASP1*: Caspase 1; *GSDMD*: Gasdermin D; *GAPDH*: Glyceraldehyde-3-phosphate dehydrogenase.

### Identification of differentially expressed genes (DEGs)

To identify DEGs in the TCGA cohort, patients were divided into two groups based on PRG expression levels: PANoptosis-high and PANoptosis-low. Differential gene expression analysis between these groups was performed using the “DESeq2” package in R (version 4.0.2) [27]. Genes with an adjusted *P* value of < 0.05 and an absolute log2 fold change greater than 1 were considered significantly differentially expressed. To reduce false positives, adjusted *P* values were used to ensure more reliable DEG selection.

### Enrichment analysis

To explore the biological functions of DEGs identified between the PRGs-high and PRGs-low cohorts, Gene Ontology (GO) and Kyoto Encyclopedia of Genes and Genomes (KEGG) enrichment analyses were performed [28, 29]. GO terms and KEGG pathways were assessed using the “clusterProfiler” package in R [30]. GO annotations encompassed molecular functions, biological processes, and cellular components, while KEGG enrichment analysis provided higher-level insights into gene function and signaling pathways. GO and KEGG analyses were conducted using corrected *P* values < 0.05 as the threshold for statistical significance. Additionally, gene set enrichment analysis (GSEA) was performed using the GSEA software (http://www.broadinstitute.org/gsea/index.jsp), focusing on the Molecular Signatures Database (MsigDB) collection, specifically c5.go.bp.v2023.2.Hs.symbols.gmt [31].

### Tumor immune analysis

The CIBERSORT algorithm (https://cibersort.stanford.edu/) was employed to evaluate the composition of the tumor immune microenvironment. Expression data from PCa samples were input into CIBERSORT, and the algorithm was executed with 1000 permutations to estimate the relative proportions of 22 distinct immune cell types [32]. The distributions of these immune cell populations in the PRGs-high and PRGs-low subgroups were determined and visualized as a landscape map. Subsequently, the ESTIMATE algorithm was applied to further characterize the TME by calculating tumor purity and immune scores, enabling a systematic pan-cancer assessment of tumor purity [33]. Furthermore, the tumor immune dysfunction and exclusion (TIDE) algorithm was used to generate TIDE scores and predict responses to immunotherapy [34].

### Somatic mutation analysis

To investigate somatic mutations in PCa samples, mutation data in “.maf” format were obtained from the TCGA Genomic Data Commons Data Portal. The “maftools” package in R was used to generate waterfall plots, providing a comprehensive depiction of the mutation landscape [35].

### Survival analysis

Kaplan–Meier (KM) survival curves were constructed using the survival and survminer packages in R to visually compare survival distributions among different patient cohorts. Log-rank tests were applied to evaluate differences in survival between these cohorts. A *P* value below 0.05 was considered statistically significant. Univariate Cox proportional hazards regression analysis was conducted to examine the relationship between risk scores and OS. Variables with a *P* value < 0.05 were deemed significant and selected for multivariate analysis. To determine whether these variables remained significant after adjusting for potential confounders, a multivariate Cox proportional hazards regression model was subsequently performed.

### Screening of prognosis-related signatures

Prognosis-related PRGs were identified through univariate Cox regression analysis. Statistically significant genes were selected as candidate inputs for further modeling. Subsequently, Lasso Cox regression analysis was performed using the glmnet package in R to calculate precise coefficient values. Based on the selected genes, a multivariate Cox model was developed [36].

### Cell culture and transient transfection

The cell lines RWPE-1, PC-3M, 22RV1, C4-2, DU145, and PC-3 were obtained from Beijing Bena Biotechnology Co. (Beijing, China). Cells were maintained in DMEM/F-12 medium supplemented with 10% fetal bovine serum (FBS) (Gibco). Transfection with either negative control (NC) or *ADAR*-targeting siRNA (Sagon, China) was performed using Lipofectamine 2000 (Invitrogen, Thermo Fisher, USA) according to the manufacturer’s protocol.

### Quantitative real-time polymerase chain reaction (qRT-PCR)

Total RNA was extracted using TRIzol reagent (Thermo Fisher, USA). Real-time PCR analysis was conducted using the FastStart Universal SYBR Green Master on 2 µg of RNA with the LightCycler 480 PCR System (Roche, USA). Each 20 µL amplification reaction included 2 µL of cDNA template, 10 µL of PCR master mix, 0.5 µL each of forward and reverse primers, and nuclease-free water to volume. The thermal cycling conditions were as follows: initial denaturation at 95 ^∘^C for 30 s, followed by 45 cycles of 94 ^∘^C for 15 s, 56 ^∘^C for 30 s, and 72 ^∘^C for 20 s. Each sample was tested in triplicate in three independent experiments. Threshold cycle (CT) values were normalized to GAPDH expression using the 2^ΔΔCT^ method. Relative mRNA expression levels were calculated in comparison to normal tissue controls.

The primer sequences used for target genes are listed in Table 1.

### Determination of 5-ethynyl-2′-deoxyuridine (EdU)

The EdU assay was conducted using the BeyoClick™ EdU Cell Proliferation Kit with Alexa Fluor 594 (Biotek, Shanghai, China). After PBS rinsing, cells were treated with EdU solution for 2 h, followed by nuclear staining with DAPI. After additional washing steps, specimens were examined under an inverted microscope (Olympus).

### Drug treatment

Docetaxel was obtained from MedChemExpress (MCE, HY-B0011), dissolved in DMSO, and added to the culture medium at the indicated concentration.

### Cell viability

Cell viability was assessed using the Cell Counting Kit-8 (CCK-8; Beyotime, China) according to the manufacturer’s protocol. Treated cells were seeded into 96-well plates at a density of 1 × 10^3^ cells per well. CCK-8 solution was added at the designated time points. After incubation for 2 h at 37 ^∘^C, the optical density at 450 nm was measured using a microplate reader (BioTek, USA).

### Immunofluorescence

Conditioned medium was collected from PC-3M and 22RV1 cell lines in both the si-NC and si-*ADAR* groups. After high-speed centrifugation, the supernatants were collected and co-cultured with THP-1-derived macrophages (Bena Biotechnology, China) to investigate the regulatory effects of *ADAR* knockdown on macrophage polarization.

After a 12-h co-culture, macrophages were fixed with paraformaldehyde for 10 min, followed by antigen blocking using QuickBlock™ solution (Beyotime, P0220). Cells were incubated overnight at 4 ^∘^C with primary antibodies: CD86 polyclonal antibody (Proteintech, 13395-1-AP) and CD206 monoclonal antibody (Proteintech, 60143-1-Ig). The next day, after a 2-h incubation at room temperature with fluorescent secondary antibodies, immunofluorescence images were captured using a Leica DM2500 microscope.

### Statistical analysis

All statistical analyses were conducted using R software (version 4.3.3) and GraphPad Prism 8. For comparisons between two cohorts with normally distributed variables, unpaired Student’s *t*-tests were used. If the data did not follow a normal distribution, the Mann–Whitney *U* test was applied. For multi-group comparisons, either parametric methods (e.g., one-way ANOVA) or non-parametric methods (e.g., Kruskal–Wallis test) were employed. KM survival analysis was performed to examine OS differences between high-risk and low-risk cohorts, with log-rank tests used to assess survival distribution. Receiver operating characteristic (ROC) curves were generated, and area under the curve (AUC) values were calculated to evaluate the predictive performance of the risk score.

**Figure 1.**
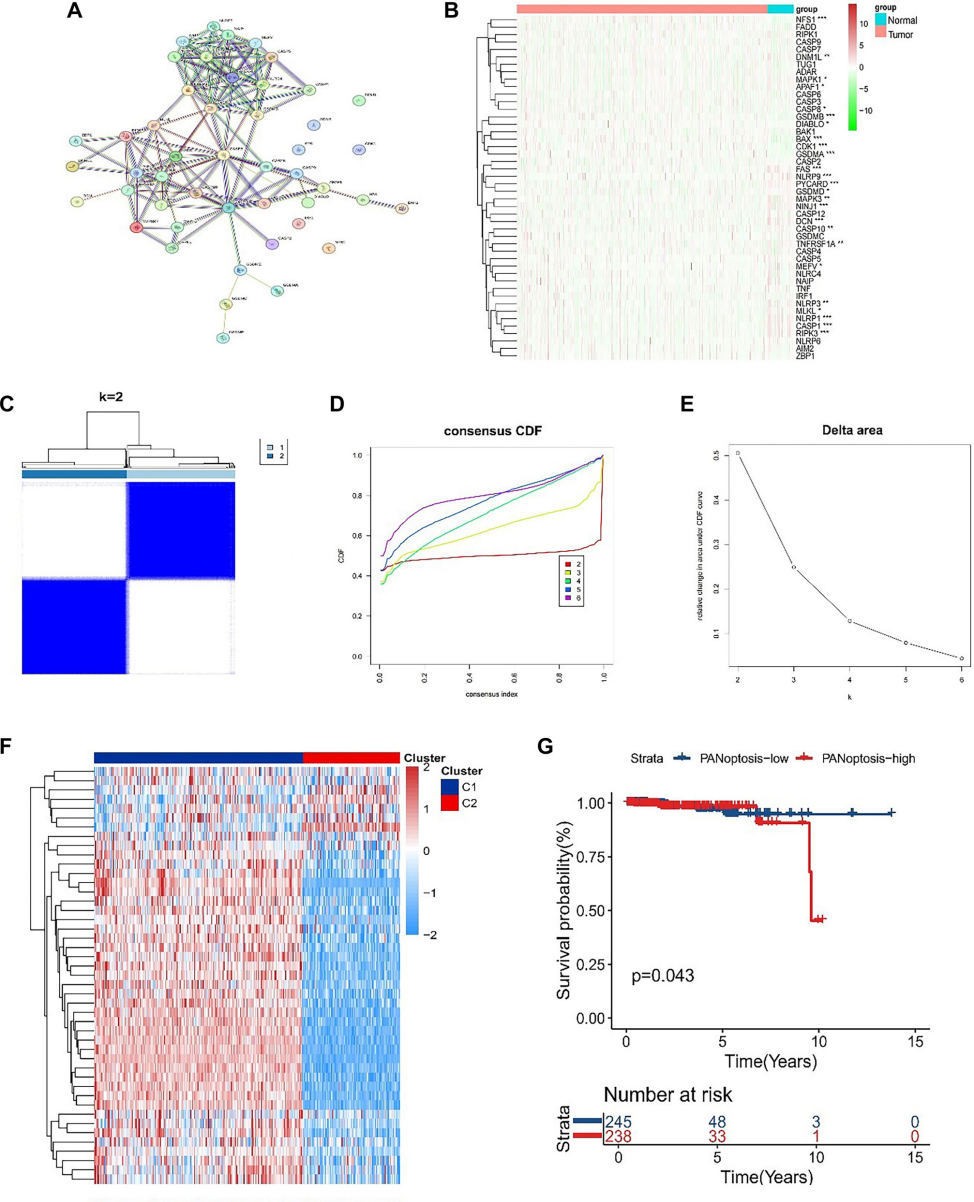
**ldentification of PANoptosis-associated subtypes.** (A) Protein-protein interaction network of PANoptosis-associated genes; (B) Heatmap showing the expression profiles of 45 PANoptosis-associated genes in normal and tumor (PCa) samples from the TCGA database; (C) Consensus clustering matrix for TCGA PCa samples at *k* ═ 2 based on the expression of 45 PANoptosis-associated genes; (D, E) Consensus cumulative distribution function (CDF) curves for *k* ═ 2–6 and the corresponding delta area plot for *k*-means consensus clustering of PANoptosis-associated genes in the TCGA PCa cohort, indicating that *k* ═ 2 provides the most stable PANoptosis-associated clustering solution; (F) Heatmap of the expression of 45 PANoptosis-associated genes in the two PANoptosis-related clusters (C1 and C2), where red denotes higher and blue lower expression levels; (G) Kaplan-Meier curves for overall survival comparing PANoptosis-high and PANoptosis-low groups in the TCGA PCa cohort. *P < 0.05, **P < 0.01, ***P < 0.001 &****P < 0.0001. Abbreviations: PCa: Prostate cancer; OS: Overall survival; PRG: PANoptosis-related gene; TCGA: The Cancer Genome Atlas; CDF: Cumulative distribution function; KM: Kaplan–Meier.

## Results

### PANoptosis-related subtypes clustering

A total of 45 PRGs were identified based on previous studies. A PPI network analysis was conducted utilizing the STRING database to uncover the interconnections among these genes (Figure 1A). Additionally, the distribution profiles of these genes were examined in both normal and PCa tissues. The findings indicated that most PRGs, including *BAX, NFS1, GSDMA, CDK1, GSDMB, DIABLO,* and *CASP8*, exhibited significant upregulation in PCa samples (Figure 1B). Furthermore, PANoptosis-associated clusters in PCa were identified through consensus clustering (Figure 1C). By implementing k-means clustering on the TCGA cohort, two clusters with distinct PRG expression patterns were distinguished (Figure 1D and 1E). Notably, cluster C2 demonstrated elevated PRG expression levels, classifying it as the PANoptosis-high subtype, whereas cluster C1 was characterized by lower expression levels, defining it as the PANoptosis-low subtype (Figure 1F). Survival analysis revealed notable differences in clinical outcomes between these subtypes, with the PANoptosis-low subtype being linked to an unfavorable prognosis, whereas the PANoptosis-high subtype exhibited better clinical results (Figure 1G).

### DEGs and enriched pathways in PANoptosis subtypes

**Figure 2.**
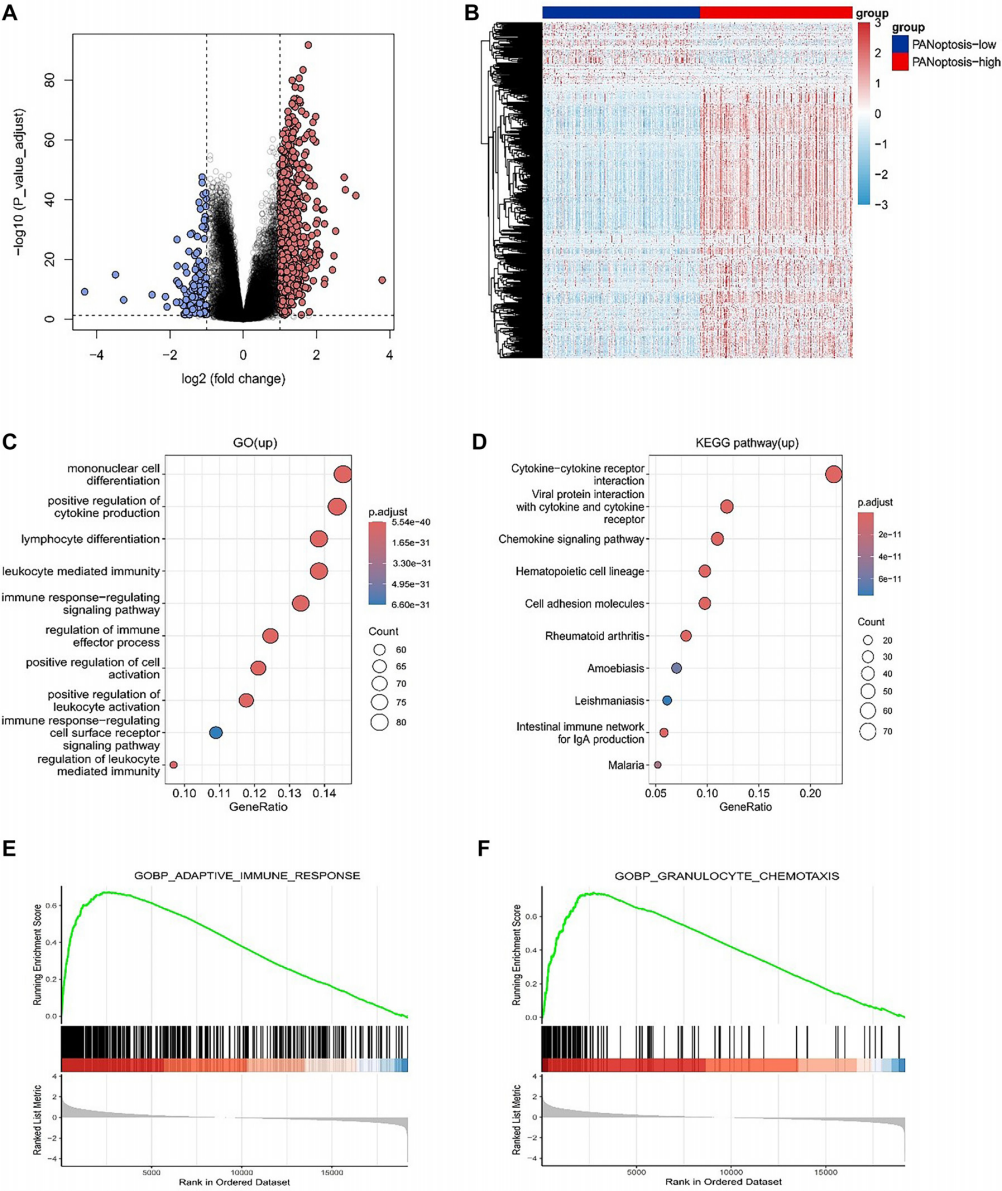
**ldentification of DEGs and associated signaling cascades across subgroups.** (A) Volcano plot of differentially expressed genes (DEGs) between PANoptosis-high and PANoptosis-low subtypes in the TCGA cohort (|log_2_ fold change| > 1 and adjusted *P* < 0.05); red and blue dots indicate up- and down-regulated genes, respectively; (B) Heatmap depicting expression patterns of DEGs across PANoptosis-high and PANoptosis-low subtypes; (C) Gene Ontology (GO) biological-process enrichment analysis of genes up-regulated in the PANoptosis-high subtype, shown as a bubble plot in which dot size represents gene count and color indicates adjusted *P* values; (D) KEGG pathway enrichment analysis of genes up-regulated in the PANoptosis-high subtype, visualized using the same bubble-plot scheme as in (C); (E–F) Gene Set Enrichment Analysis (GSEA) based on ranked gene expression between PANoptosis-high and PANoptosis-low subtypes, illustrating enrichment of representative immune-related GO biological processes, including adaptive immune response (E) and granulocyte chemotaxis (F). Abbreviations: TCGA: The Cancer Genome Atlas; DEG: Differentially expressed gene; GO: Gene Ontology; KEGG: Kyoto Encyclopedia of Genes and Genomes; GSEA: Gene set enrichment analysis.

Considering that the PANoptosis-high subtype was linked to favorable clinical outcomes, whereas the PANoptosis-low subtype was associated with an unfavorable prognosis, an investigation was conducted to identify key DEGs and signaling pathways in each subtype to elucidate the molecular mechanisms influencing prognosis. The analysis identified 746 DEGs (Figure 2A and 2B), encompassing 617 upregulated and 129 downregulated genes.

GO enrichment analysis demonstrated that elevated genes within the PANoptosis-high subtype were predominantly involved in immune-related biological processes, encompassing cytokine production enhancement, lymphocyte differentiation, leukocyte-mediated immunity, and immune response-regulating signaling pathways. Similarly, KEGG pathway enrichment analysis suggested that these elevated genes were markedly associated with immune activity pathways, including cytokine–cytokine receptor interaction, viral protein interaction with cytokine and cytokine receptors, and chemokine signaling (Figure 2C). Collectively, these findings suggest that the PANoptosis-high subtype is defined by an immune-active microenvironment, which may contribute to its more favorable clinical outcomes. To examine the activated signaling cascades within the PANoptosis-high subgroup, GSEA was conducted to compare the PANoptosis-high and PANoptosis-low cohorts. This analysis identified differential enrichment of gene sets linked to immune pathways, including granulocyte chemotaxis and the adaptive immune response (Figure 2D).

### Somatic mutations and TME in PANoptosis subtypes

**Figure 3.**
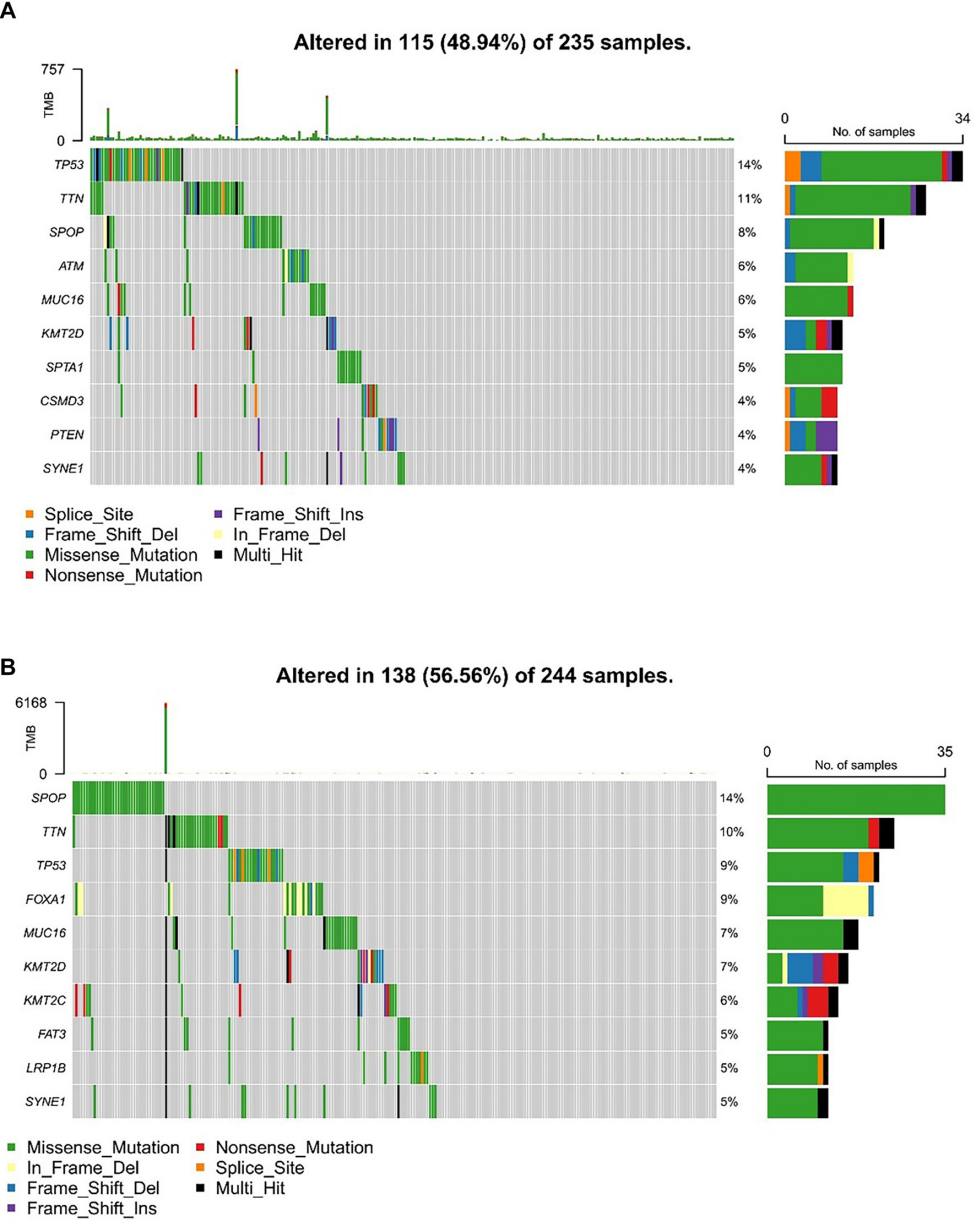
**Analysis of somatic alterations across distinct PANoptosis subtypes.** (A and B) Graphs showing the top ten genes with the highest mutation frequencies in the PANoptosis-high (A) and PANoptosis-low (B) subtypes.

Distinct somatic mutation profiles were identified across the subtypes (Figure 3A and 3B). Although *TP53, TTN, SPOP, MUC16*, and *SYNE1* emerged as the genes with the highest mutation rates, their mutation frequencies differed between subtypes. Notably, the PANoptosis-high subtype demonstrated a higher prevalence of *TP53* and *TTN* mutations, comprising 14% and 11% of total mutations, respectively, in contrast to 9% and 10% observed in the PANoptosis-low subtype.

**Figure 4.**
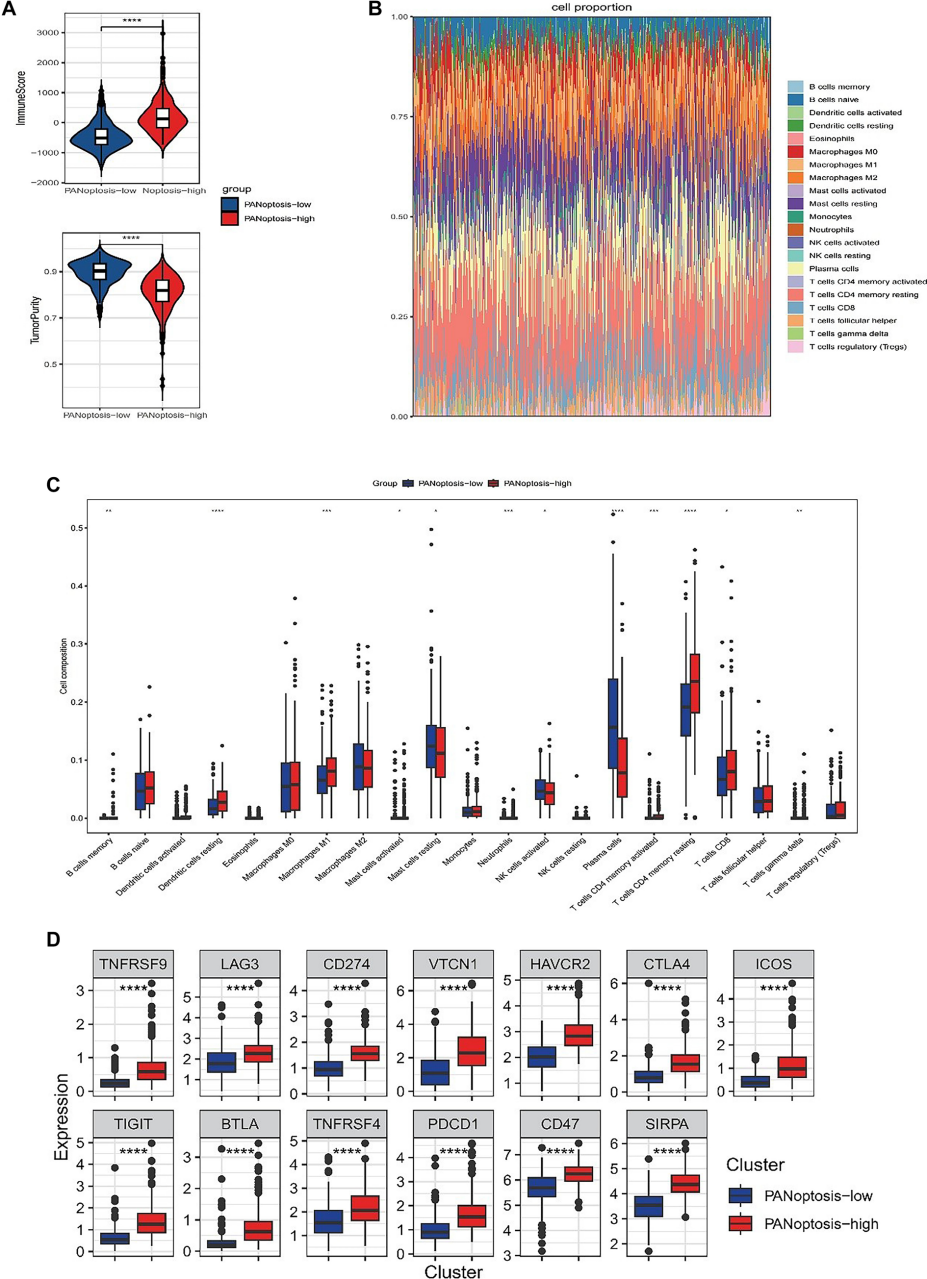
**Immune landscape of PANoptosis-high and PANoptosis-low subtypes.** (A) Violin plots compare ESTIMATE-derived ImmuneScore and tumor purity between PANoptosis-high and PANoptosis-low subtypes; (B) Composition plot showing the relative fractions of tumor-infiltrating immune cell subsets in each sample from the PANoptosis-high and PANoptosis-low subtypes; (C) Box plots compare the abundance of representative immune cell subsets between PANoptosis-high and PANoptosis-low subtypes; (D) Box plots display the differential expression of multiple immune checkpoint-related genes in PANoptosis-high and PANoptosis-low subtypes. *P < 0.05, **P < 0.01, ***P < 0.001, &****P < 0.0001.

Growing research indicates that PANoptosis plays a vital role in eliciting anti-tumor immune responses. This investigation analyzed the TME configuration concerning PANoptosis-high and PANoptosis-low classifications. The analysis revealed that immune scores were markedly elevated, while tumor purity was notably reduced in the PANoptosis-high category relative to the PANoptosis-low cohort (Figure 4A).

The investigation further explored variations in immune cell infiltration across 22 immune cell populations between these classifications utilizing the CIBERSORT methodology combined with the LM22 signature matrix. The outcomes obtained from examining 544 PCa cases within the TCGA database are depicted in Figure 4B. Notably, patients classified under the PANoptosis-high subtype demonstrated a substantially increased presence of memory B cells, resting dendritic cells, M1 macrophages, activated mast cells, neutrophils, activated memory CD4^+^ T cells, resting memory CD4^+^ T cells, CD8^+^ T cells, and γ δ T cells (Figure 4C).

Furthermore, all immune checkpoints exhibited upregulation in the PANoptosis-high subtype, whereas a contrasting trend was detected in the PANoptosis-low subtype (Figure 4D). This finding suggests that the PANoptosis-high subtype is linked to an immune-hot phenotype, while the PANoptosis-low subtype corresponds to an immune-cold phenotype.

### Development and verification of the PANoptosis risk signature

**Figure 5.**
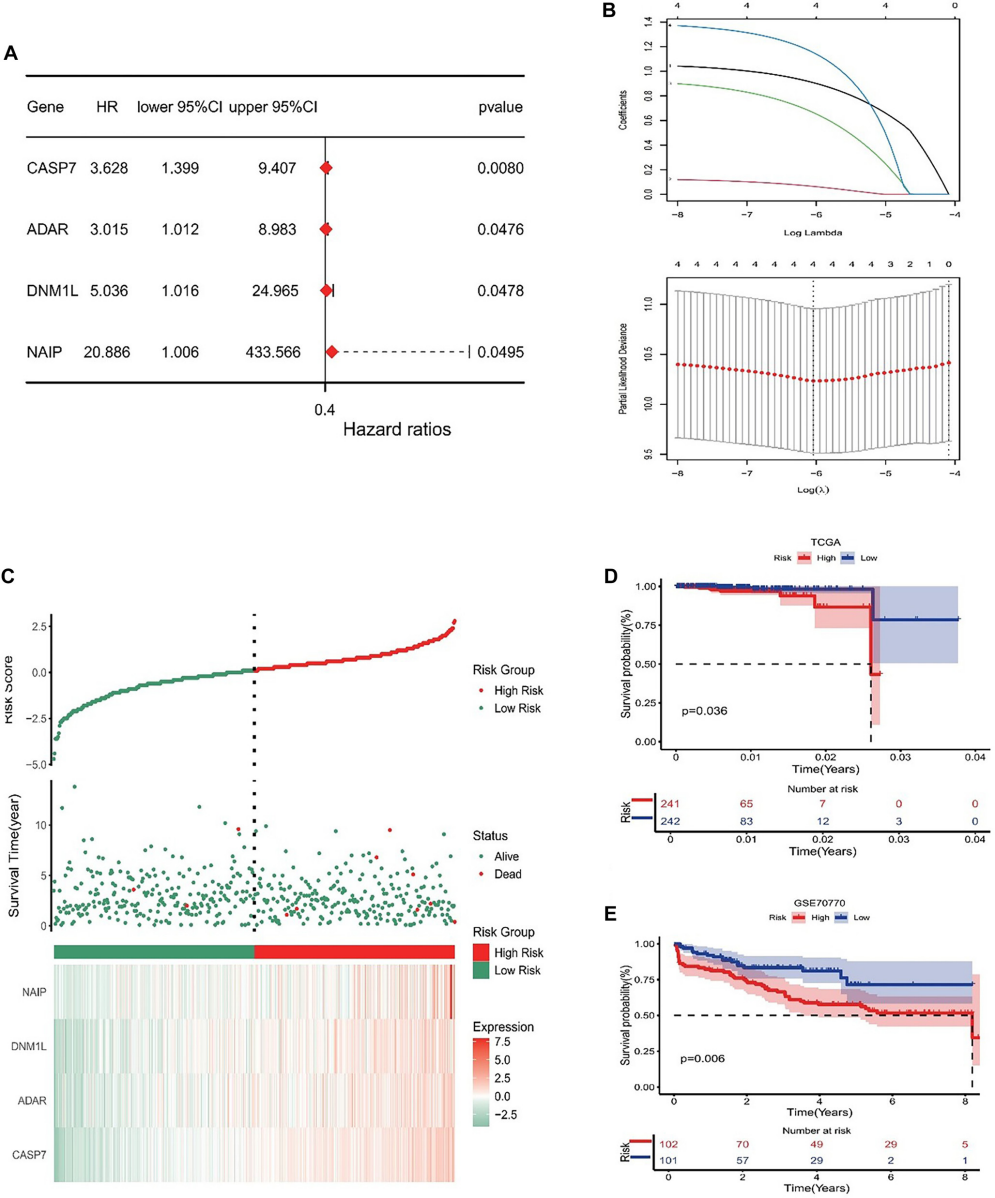
**Construction and validation of the PANoptosis risk signature.** (A) The prognostic value of PANoptosis-related genes for OS is assessed using univariate Cox analysis; (B) Lasso-Cox regression identifies four genes most closely associated with OS in the TCGA dataset; (C) The distribution of risk scores, patient survival status, and heatmaps representing the prognostic four-gene signature within the TCGA database; (D and E) KM analyses demonstrate the prognostic relevance of the risk model in the TCGA and GSE70770 cohorts. Abbreviations: OS: Overall survival; TCGA: The Cancer Genome Atlas; KM: Kaplan–Meier.

A prognostic model was developed based on PRGs. In the Cox univariate analysis, four PRGs were identified as being markedly linked to OS in patients (Figure 5A). These four PRGs were selected and validated for inclusion in the prediction model using LASSO regression analysis (Figure 5B). The risk score model was formulated using the following equation: Risk score ═ (0.9075)*CASP7 + (0.0645)*ADAR + (0.6644)*DNM1L + (1.1522)*NAIP. Additionally, the correlation between survival status and risk score was investigated. These observations indicated that the proportion of surviving patients in the low-risk cohort was markedly higher than in the high-risk cohort (Figure 5C). The prognostic relevance of this risk profile in PCa was further assessed through KM analysis (Figure 5D). Within the TCGA cohort, a high-risk score was linked to poorer OS, and these outcomes were validated in the GEO dataset (Figure 5E).

**Figure 6.**
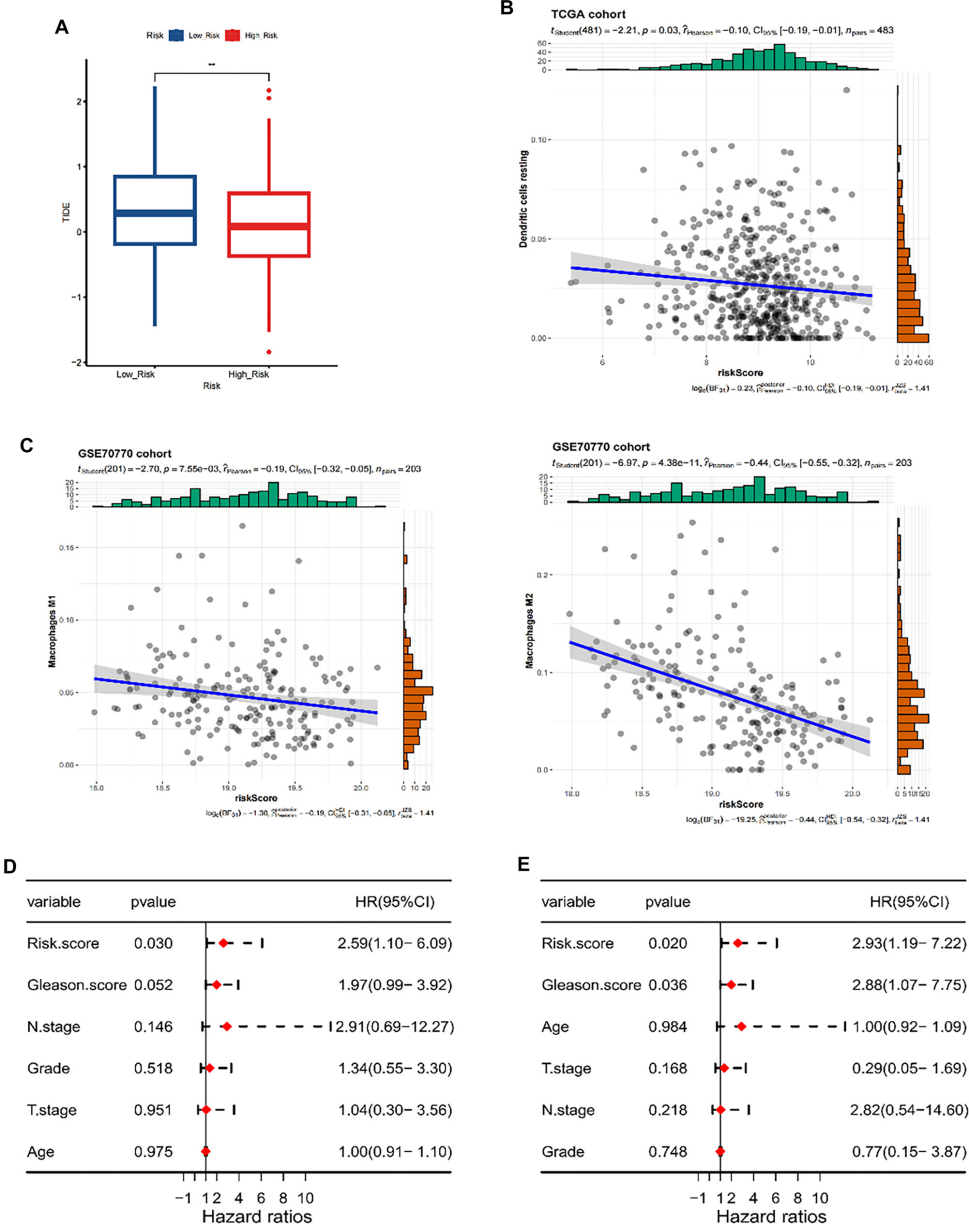
**Association between the PANoptosis risk signature and the TME.** (A) Box plot comparing TIDE scores between PANoptosis-based low-risk and high-risk groups, reflecting predicted response to immune checkpoint blockade; (B) Scatter plot showing the correlation between the PANoptosis risk score and the ESTIMATE stromal score in the TCGA cohort; (C) Scatter plots depict the association between the PANoptosis risk score and macrophage infiltration (M1 and M2) in the GSE70770 cohort; (D, E) Univariate (D) and multivariate (E) Cox regression analyses evaluating the independent prognostic value of the PANoptosis-based risk score alongside clinicopathological variables in patients with prostate cancer. Abbreviations: PCa: Prostate cancer; TCGA: The Cancer Genome Atlas; TME: Tumor microenvironment.

### Connection between PANoptosis risk signature and TME

The TIDE tool was employed to assess the predictive potential of the PANoptosis risk signature in determining the clinical efficacy of immunotherapy. The findings indicated that patients classified in the immunotherapy non-response cohort exhibited higher PANoptosis risk scores, suggesting that those with lower risk scores might derive greater benefit from immunotherapy (Figure 6A).

**Figure 7.**
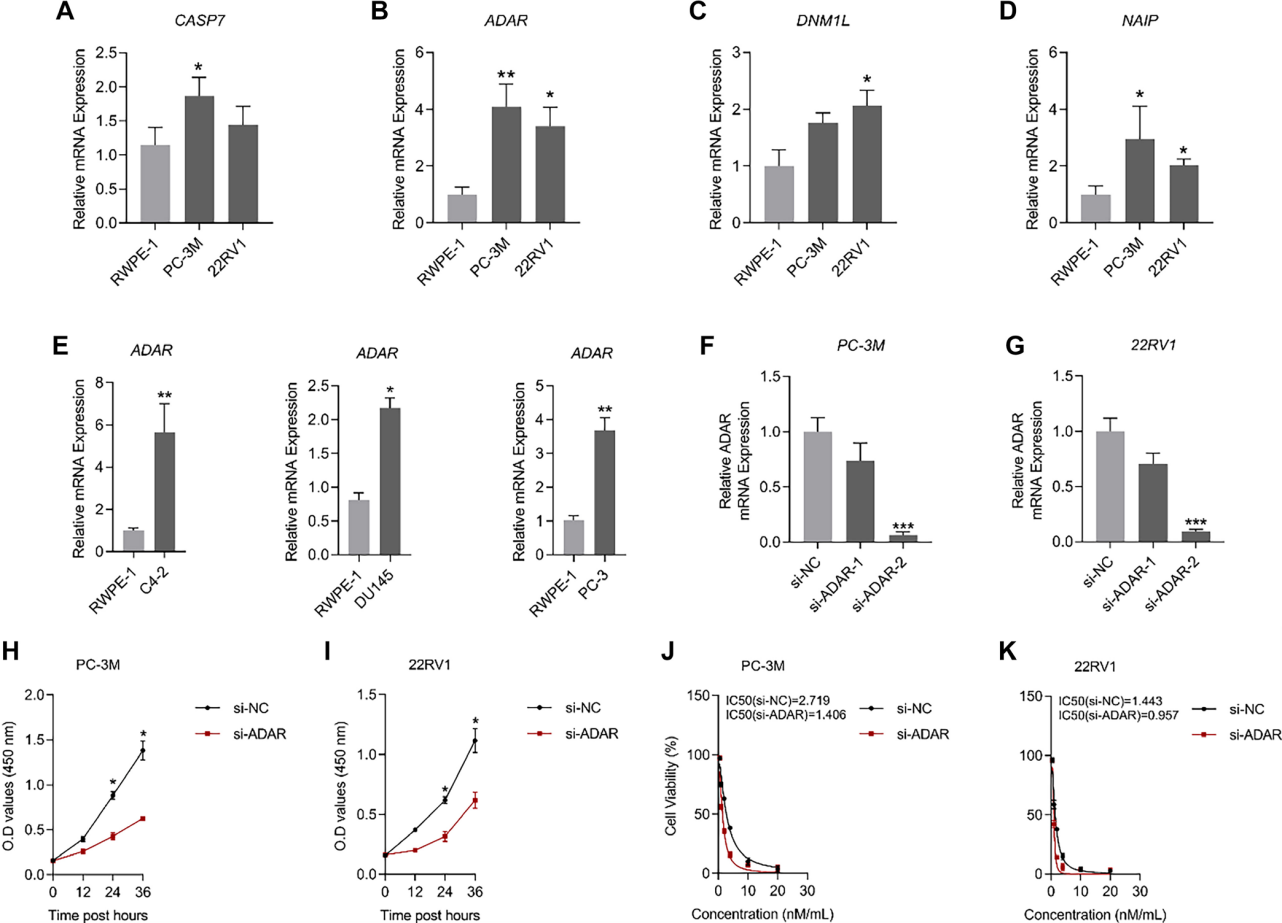
***ADAR* is upregulated in PCa cell lines.** (A–D) Transcript levels of *CASP7, ADAR, DNM1L*, and *NAIP* were analyzed by qRT-PCR in RWPE-1, PC-3M, and 22RV1 cell lines; (E) qRT-PCR was performed to assess *ADAR* transcript levels in RWPE-1, C4-2, DU145, and PC-3 cell lines; (F and G) Knockdown efficiency of siRNAs targeting *ADAR* in PC-3M and 22RV1 was evaluated by qRT-PCR; (H and I) Changes in PC-3M and 22RV1 cell viability before and after *ADAR* silencing were measured; (J and K) IC50 variations in PC-3M and 22RV1 were assessed following Docetaxel treatment before and after *ADAR* knockdown. *n* ═ 3/per cohort. Abbreviations: PCa: Prostate cancer; qRT-PCR: Quantitative real-time polymerase chain reaction.

Considering PANoptosis’s crucial immunological function in anti-tumor immune responses, an in-depth analysis was performed to investigate the association between the PANoptosis risk score and the TME. The outcomes demonstrated that an elevated risk score was negatively associated with resting dendritic cells (Figure 6B). Additionally, in the GEO cohort, a negative correlation was observed between an increased risk score and both Macrophages M1 and Macrophages M2 (Figure 6C).

Univariate and multivariate Cox analyses were performed to assess the independent prognostic significance of the PANoptosis risk signature. The univariate Cox analysis identified a high PANoptosis risk score as significantly associated with diminished OS (Figure 6D). Moreover, multivariate Cox analysis confirmed the PANoptosis risk score’s capability to serve as an independent prognostic factor for individuals with PCa (Figure 6E).

### *ADAR* promotes PCa progression *in vitro*

To validate the bioinformatics model, *CASP7, ADAR, DNM1L*, and *NAIP* transcript levels were analyzed in PCa cell lines. Compared to RWPE-1, transcripts of *CASP7, ADAR, DNM1L*, and *NAIP* were upregulated in PC-3M and 22RV1, with *ADAR* demonstrating the most pronounced increase (Figure 7A–7D). Furthermore, *ADAR* transcript levels in RWPE-1 were evaluated against those in C4-2, DU145, and PC-3. *ADAR* was markedly upregulated in the PCa cell lines C4-2, DU145, and PC-3, indicating that *ADAR* transcript upregulation is a widespread phenomenon in PCa cell lines (Figure 7E).

For this reason, *ADAR* was selected for further investigation. The inhibitory efficiency of small interfering RNAs targeting *ADAR* was validated in PC-3M and 22RV1. Both *si-ADAR-1* and *si-ADAR-2* suppressed *ADAR* transcription in these cell lines, with *si-ADAR-2* exhibiting the most pronounced inhibitory effect (Table 2). Consequently, *si-ADAR-2* (*si-ADAR*) was chosen for subsequent experiments (Figure 7F and 7G). A substantial reduction in cell viability was observed after *ADAR* suppression in PC-3M and 22RV1 cell lines. Additionally, the IC50 values of PC-3M and 22RV1 treated with Docetaxel *in vitro* were markedly decreased (Figure 7H–7K).

**Table 2:** Target sequences (5′–3′) of siRNAs directed against ADAR

**Gene**	**Target sequences (5′–3′)**
*si-ADAR-1*	CAGTAGTTTCCTGCTTAAGCAAA
*si-ADAR-2*	CTGCGACTATCTCTTCAATGTGT

Abbreviations: *siRNA*: Small interfering RNA; *ADAR*: Adenosine deaminase acting on RNA.

**Figure 8.**
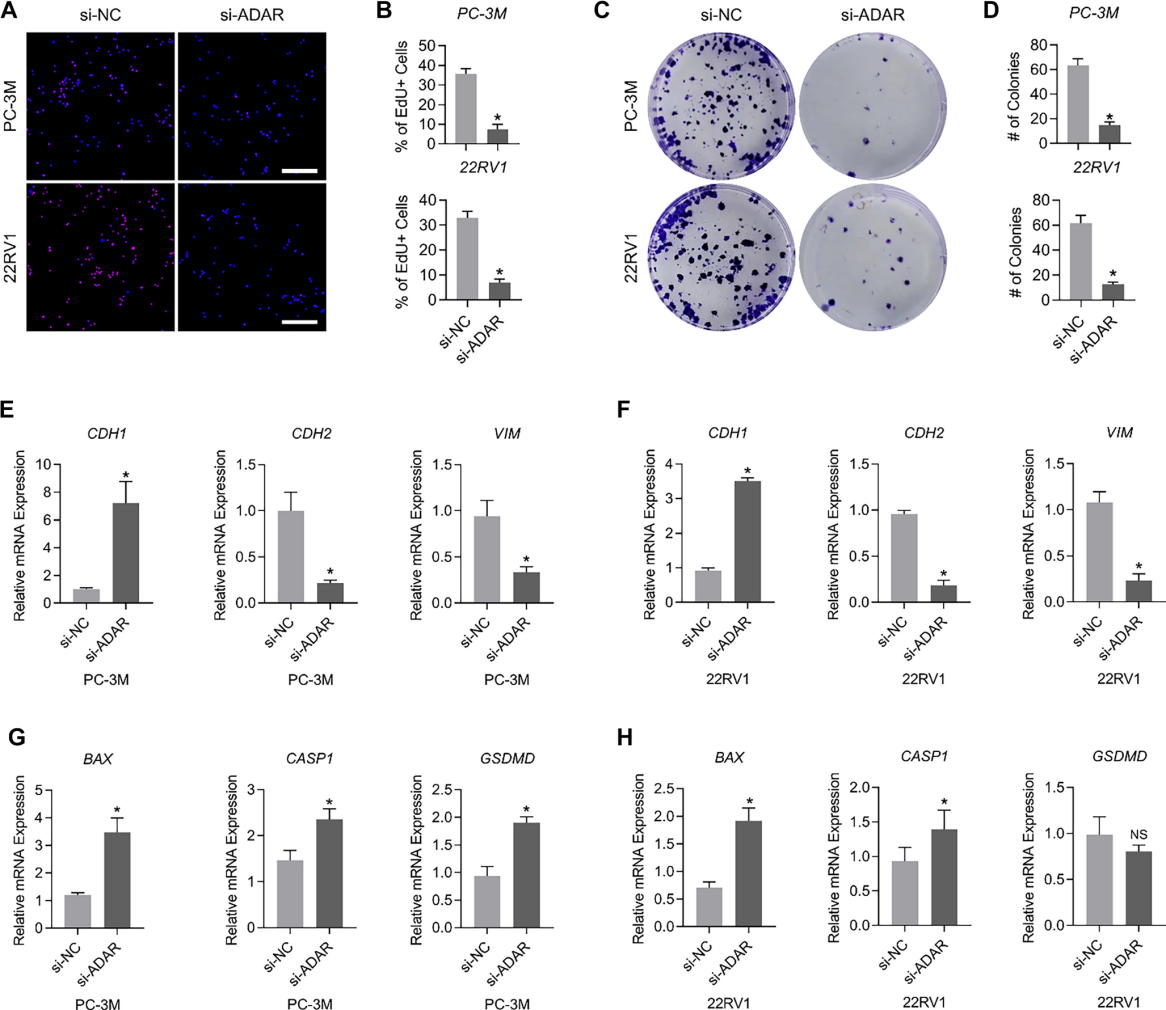
***ADAR* promotes PCa progression *in vitro*.** (A and B) Changes in the proportion of EdU-positive cells in PC-3M and 22RV1 before and after *ADAR* knockdown; (C and D) Alterations in colony formation in PC-3M and 22RV1 before and after *ADAR* knockdown; (E and F) Changes in the expression of *CDH1, CDH2*, and *VIM* in PC-3M and 22RV1 before and after *ADAR* knockdown; (G and H) Changes in the expression of *BAX, CASP1*, and *GSDMD* in PC-3M and 22RV1 after *ADAR* knockdown. *n* ═ 3/per cohort. PCa: Prostate cancer; EdU: 5-ethynyl-2′-deoxyuridine.

The proliferative capacity of PC-3M and 22RV1 cell lines was markedly reduced following *ADAR* knockdown, as indicated by a decline in the percentage of EdU-positive cells and a reduction in colony formation (Figure 8A–8D). Given that EMT plays a pivotal role in cancer metastasis, the transcription of EMT-related markers in PC-3M and 22RV1 was examined before and after *ADAR* knockdown. The results demonstrated that CDH1 expression was upregulated, whereas *CDH2* and *VIM* were downregulated following *ADAR* silencing (Figure 8E and 8F), suggesting that *ADAR* promotes EMT in PCa cell lines.

Additionally, the impact of *ADAR* on cellular apoptosis and pyroptosis was assessed, revealing that the transcriptional upregulation of *BAX, CASP1*, and *GSDMD* following *ADAR* knockdown reflects its inhibitory effect on programmed cell death (Figure 8G and 8H).

**Figure 9.**
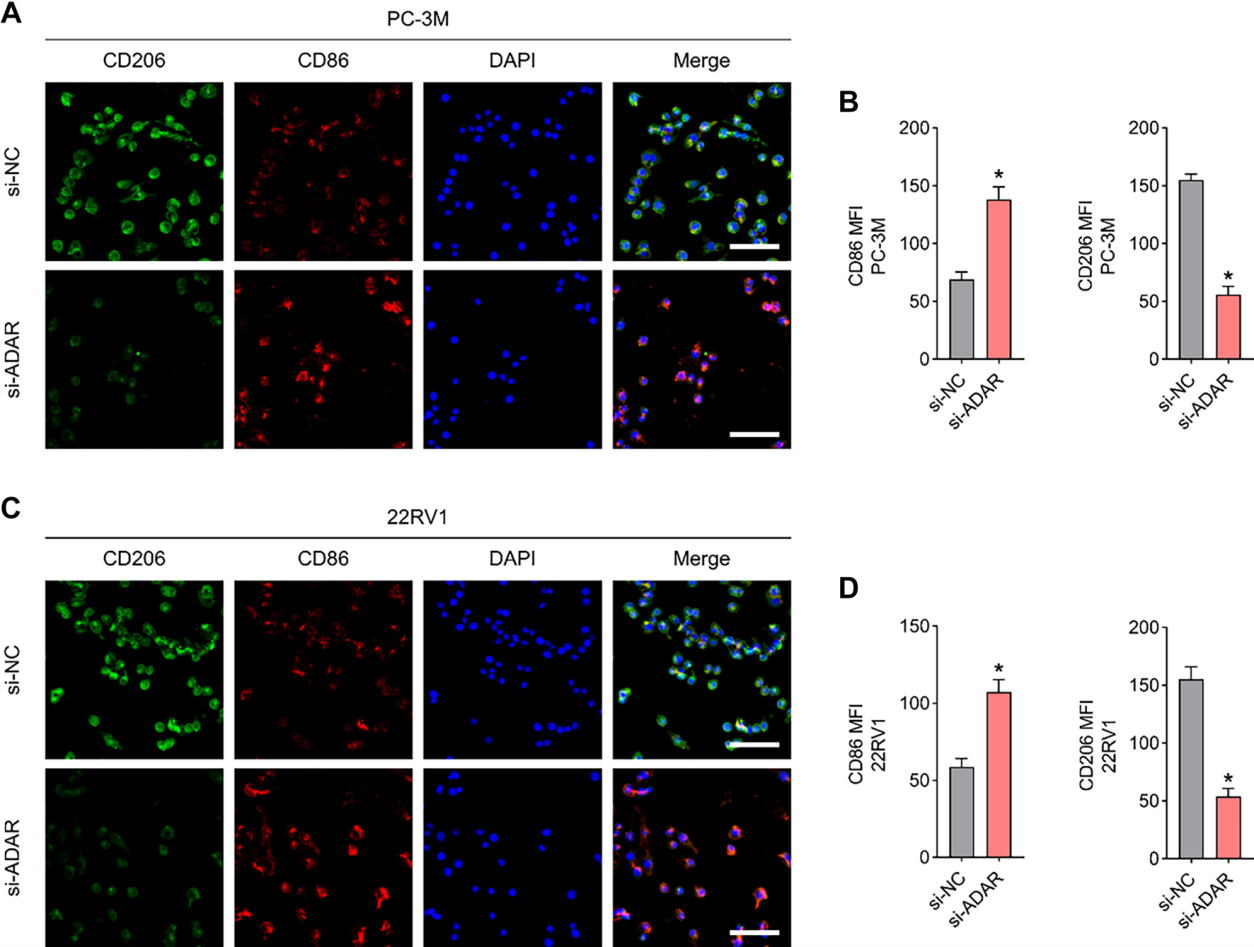
***ADAR* modulates macrophage polarization.** (A–D) Changes in surface expression levels of CD86 and CD206 in macrophages before and after *ADAR* knockdown. *n* ═ 3/per cohort.

Finally, the regulatory influence of *ADAR* on the immune microenvironment was examined through its impact on macrophages, which tended to polarize toward the M1 phenotype following *ADAR* knockdown. This observation suggests that *ADAR* contributes to establishing an inflammation-suppressive TME (Figure 9A–9D).

## Discussion

Despite notable advancements in diagnostic and therapeutic methodologies, PCa continues to be a predominant contributor to cancer-related mortality in men, with recurrence occurring in up to 40% of individuals diagnosed with localized PCa within a decade [37–39]. Androgen deprivation therapy has long been recognized as a conventional approach for PCa treatment. However, a considerable proportion of patients with advanced-stage disease inevitably progress to castration-resistant PCa [40]. In recent years, innovative treatment approaches, including immunotherapy, have been investigated to potentiate the immune system’s capacity to eradicate malignant cells [41–44]. Nevertheless, the effectiveness of immunotherapy in managing PCa, as well as its implications for patient prognosis, remains a topic of ongoing debate. Certain studies indicate that the intricate TME intrinsic to PCa fosters an inherent resistance to immunotherapeutic interventions, thereby complicating the identification of efficacious treatment strategies [45–47]. Consequently, delineating subpopulations that benefit from PCa immunotherapy and establishing biomarkers predictive of patient prognosis are imperative steps toward optimizing therapeutic outcomes in PCa management.

Recent investigations have highlighted the essential role of PRGs in PCa [48–50]. These genes govern the assembly of the PANoptosome complex, which integrates the molecular mechanisms underlying pyroptosis, necroptosis, and apoptosis [7]. This unified pathway, termed PANoptosis, is pivotal in modulating tumor cell death and immune responses in PCa. Aberrant expression and notable mutations in PRGs have been identified across multiple cancer types, with numerous PRGs functioning as tumor risk determinants in diverse malignancies. These observations indicate that PANoptosis serves a crucial function in cancer development, and its induction could potentially suppress both tumor initiation and progression [17]. For instance, research conducted by Jianzhong et al. demonstrates that modifications in PRG expression can substantially alter the immune microenvironment and therapeutic responsiveness in PCa [51]. Similarly, research led by Wang et al. [52] identified a notable connection between PANoptosis and clear cell renal cell carcinoma, further establishing a prognostic model utilizing three specific miRNAs to predict survival outcomes in cancer patients. Within the scope of PRAD research, the intricate interactions and regulatory networks linked to PANoptosis have been explored, encompassing gene mutations, transcriptional alterations, methylation modifications, and their correlations with clinical characteristics [17]. Hence, this investigation sought to understand PANoptosis functionality in PCa and construct a prognostic signature based on PRGs.

In this study, a total of 45 PRGs were compiled from previously conducted relevant research. Based on the expression profiles of these PRGs, PCa patients were categorized into two distinct PANoptosis subgroups through consensus clustering. Subsequently, differential gene expression and functional enrichment analyses were performed, revealing that these genes participate in immune-associated pathways, including the PI3K-Akt and TNF signaling pathways. To further delineate immune disparities between the PANoptosis-high and PANoptosis-low subgroups and deepen the understanding of the TME’s role in disease progression and treatment outcomes, immune infiltration and TIDE analyses were conducted. The findings indicate a substantial divergence in immune phenotypes: the PANoptosis-high subgroup exhibits an immune-hot phenotype, whereas the PANoptosis-low subgroup presents an immune-cold phenotype. In individuals classified within the PANoptosis-high subgroup, an increased proportion of resting dendritic cells, M1 macrophages, and other immune cells was observed.

The TME has been shown to have a significant impact on tumors [53–56]. Tumor-associated macrophages (TAMs) constitute a major immune cell population within the inflammatory TME. As heterogeneous macrophages, TAMs exhibit both pro-inflammatory (M1) and immunosuppressive (M2) functionalities [57]. M1 macrophages are pivotal in the PCa TME, characterized by their ability to secrete abundant pro-inflammatory cytokines and chemokines. These cells demonstrate an enhanced capacity for antigen presentation and complement-mediated phagocytosis, primarily functioning to eliminate pathogens and initiate Th1 immune responses while also exerting direct cytotoxic effects on both microorganisms and tumor cells. Conversely, M2 macrophages are commonly implicated in tumor immune evasion, angiogenesis, tumor proliferation, and metastasis [58]. The polarization of macrophages is a highly intricate biological process meticulously regulated by multiple factors, with its polarization state exerting a profound influence on inflammatory responses and tumor development [58].

Within the complex TME of PCa, various chemokines modulate macrophage polarization. For instance, CCL2 has been shown to enhance LPS-induced IL-10 production, while CCL2 inhibition stimulates the expression of genes and cytokines associated with M1 polarization and concurrently suppresses M2 markers [59]. Additionally, research by Cristina I. Caescu and colleagues identified miR-21 as a molecule activated by the Y721 site (pTyr-721) of colony-stimulating factor-1 (CSF-1), which facilitates suppression of the M1 phenotype while promoting the M2 phenotype [60]. Another critical regulatory factor, miRNA-155, exhibits significant elevation during M1 macrophage polarization and a notable reduction during M2 polarization. Enhanced expression of miRNA-155 promotes the polarization of M2 macrophages through the miR-155/SHIP1 pathway, consequently expediting tumor cell invasion, proliferation, and migration [61, 62]. Based on these findings, it is postulated that M1 macrophage infiltration may provide survival advantages for patients exhibiting high PANoptosis subtypes. Further investigation into the regulatory mechanisms governing macrophage polarization in PCa could yield novel insights and therapeutic strategies. Subsequent survival analysis identified four PRGs—*CASP7, ADAR, DNM1L*, and *NAIP*—as being markedly associated with PCa prognosis. Cysteine aspartate-specific proteases (caspases) serve crucial functions in apoptosis and inflammatory regulation, with *CASP7* (Caspase-7) being a key protease modulating these processes [63, 64]. Research by So Hee Kim et al. demonstrated that OTUD6A functions as a deubiquitinase by eliminating the K48-linked polyubiquitin chain from nucleolin and the K63-linked polyubiquitin chain from Caspase-7. Both nucleolin and Caspase-7, identified as OTUD6A substrates, have been proposed as potential therapeutic targets in cancer treatment [65].

*ADAR* is a core enzyme involved in RNA editing [66], with previous research indicating a strong correlation between *ADAR* dysregulation and tumor onset and progression. Julia Ramírez-Moya et al. found that *ADAR* promotes thyroid cancer development through RNA editing of *CDK13* [67]. Similarly, Yu et al. reported that *ADAR* is markedly upregulated in bladder cancer tissues and shows a strong correlation with unfavorable patient outcomes. Furthermore, *ADAR* has been shown to markedly enhance the proliferation, migration, and invasion of bladder cancer cells [68].

*DNM1L*, a mitochondrial fission-associated protein, has emerged as a promising therapeutic candidate across various malignancies [69]. Research by Inoue Yamauchi et al. [70] revealed that the absence of *DNM1L* increases apoptosis in colon cancer cells. Additionally, Xie et al. [71] discovered that *DNM1L* depletion induces apoptosis in malignant brain tumor-initiating cells and markedly inhibits tumor growth.

*NAIP*, which belongs to the IAP family, is expressed in mammalian cells and can inhibit apoptosis triggered by diverse signals [72]. Moreover, previous studies have suggested that *NAIP* mediates innate immune inflammatory responses by activating caspase-1, -4, and -5 [73]. A study conducted by Chen et al. [74] demonstrated that *NAIP* alleles undergo methylation in normal oral mucosal tissue, potentially representing an early oncogenic event. Additionally, Choi et al. [75] observed that *NAIP* expression is absent in normal breast tissue but is markedly elevated in breast cancer. Although these genes have been extensively associated with the pathogenesis and progression of various malignancies, their specific mechanistic roles in PCa remain to be fully elucidated. Ultimately, prognosis-associated signatures derived from PRGs exhibited strong predictive performance for OS across training, internal validation, and external validation cohorts.

Despite the promising outcomes observed, certain limitations inherent to this research must be acknowledged. Firstly, while PRG-based prognostic features demonstrated strong predictive efficacy across both internal and external cohorts, the study predominantly relies on retrospective data, which may introduce potential biases. To confirm the clinical applicability and robustness of the proposed risk model, prospective clinical trials remain essential. Secondly, although functional enrichment and immune infiltration analyses offer valuable insights into the TME and the immune disparities between PANoptosis-high and PANoptosis-low subgroups, the precise molecular mechanisms underlying these differences have yet to be fully elucidated. Addressing these limitations in future investigations will further reinforce the clinical significance of PRGs and their potential role in informing PCa treatment strategies.

## Conclusion

In conclusion, a practical PANoptosis-risk algorithm based on four PRGs was developed and validated, with the proposed signature serving as a potential prognostic model for PCa. Furthermore, this study offers novel insights into the relationship between the PRG score and the immune microenvironment, providing valuable perspectives for the application of immunotherapy in PCa patients.

## Supplemental data

**Table S1:** List of PANoptosis-related genes

*CASP10*	*CASP2*	*CASP3*
*DCN*	*APAF1*	*CASP7*
*FAS*	*ZBP1*	*GSDMA*
*CASP8*	*IRF1*	*FADD*
*TNFRSF1A*	*RIPK3*	*MLKL*
*GSDMB*	*NINJ1*	*CDK1*
*BAX*	*CASP9*	*NLRP6*
*DNM1L*	*RIPK1*	*DIABLO*
*NLRC4*	*CASP1*	*NLRP9*
*NLRP1*	*CASP5*	*CASP4*
*MAPK1*	*CASP6*	*CASP12*
*MAPK3*	*GSDMC*	*TNF*
*MEFV*	*ADAR*	*NFS1*
*PYCARD*	*NLRP3*	*NAIP*
*GSDMD*	*AIM2*	*TUG1*

Abbreviations: *CASP10*: Caspase 10; *DCN*: Decorin; *CASP8*: Caspase 8; *TNFRSF1A*: Tumor necrosis factor receptor superfamily member 1A; *GSDMB*: Gasdermin B; *BAX*: Bcl-2-associated X protein; *NLRP1*: NLR family pyrin domain containing 1; *MAPK1*: Mitogen-activated protein kinase 1; *MAPK3*: Mitogen-activated protein kinase 3; *GSDMD*: Gasdermin D; *CASP2*: Caspase 2; *APAF1*: Apoptotic protease activating factor 1; *ZBP1*: Z-DNA binding protein 1; *IRF1*: Interferon regulatory factor 1; *NINJ1*: Ninjurin 1; *CASP9*: Caspase 9; *RIPK1*: Receptor-interacting serine/threonine-protein kinase 1; *CASP1*: Caspase 1; *CASP5*: Caspase 5; *CASP6*: Caspase 6; *GSDMC*: Gasdermin C; *ADAR*: Adenosine deaminase acting on RNA; *CASP3*: Caspase 3; *CASP7*: Caspase 7; *GSDMA*: Gasdermin A; *CDK1*: Cyclin-dependent kinase 1; *NLRP6*: NLR family pyrin domain containing 6; *NLRP9*: NLR family pyrin domain containing 9; *CASP4*: Caspase 4; *CASP12*: Caspase 12; *TNF*: Tumor necrosis factor; *TUG1*: Taurine upregulated gene 1.

## Data Availability

The training set data was sourced from the TCGA database (https://portal.gdc.cancer.gov). Corresponding clinical information for the training cohort was retrieved from the UCSC Xena database (https://xenabrowser.net/datapages/). Data for the external validation set (GSE70770) was obtained from the GEO database (https://www.ncbi.nlm.nih.gov/geo/)

## References

[1] Rebello RJ, Oing C, Knudsen KE, Loeb S, Johnson DC, Reiter RE (2021). Prostate cancer. Nat Rev Dis Primers.

[2] Parker C, Castro E, Fizazi K, Heidenreich A, Ost P, Procopio G (2020). Prostate cancer: ESMO clinical practice guidelines for diagnosis, treatment and follow-up. Ann Oncol.

[3] Siegel DA (2020). Prostate cancer incidence and survival, by stage and race/ethnicity—United States, 2001–2017. MMWR.

[4] Litwin MS, Tan HJ (2017). The diagnosis and treatment of prostate cancer: a review. JAMA.

[5] Nguyen-Nielsen M, Borre M (2016). Diagnostic and therapeutic strategies for prostate cancer. Semin Nucl Med.

[6] Sun X, Yang Y, Meng X, Li J, Liu X, Liu H (2024). PANoptosis: mechanisms, biology, and role in disease. Immunol Rev.

[7] Zhu P, Ke ZR, Chen JX, Li SJ, Ma TL, Fan XL (2023). Advances in mechanism and regulation of PANoptosis: prospects in disease treatment. Front Immunol.

[8] Pandian N, Kanneganti TD (2022). PANoptosis: a unique innate immune inflammatory cell death modality. J Immunol.

[9] Shi C, Cao P, Wang Y, Zhang Q, Zhang D, Wang Y (2023). PANoptosis: a cell death characterized by pyroptosis, apoptosis, and necroptosis. J Inflamm Res.

[10] Karati D, Kumar D (2024). Molecular insight into the apoptotic mechanism of cancer cells: an explicative review. Curr Mol Pharmacol.

[11] Qi Z, Zhu L, Wang K, Wang N (2023). PANoptosis: Emerging mechanisms and disease implications. Life Sci.

[12] Ocansey DKW, Qian F, Cai P, Ocansey S, Amoah S, Qian Y (2024). Current evidence and therapeutic implication of PANoptosis in cancer. Theranostics.

[13] Liu Z, Sun L, Peng X, Zhu J, Wu C, Zhu W (2024). PANoptosis subtypes predict prognosis and immune efficacy in gastric cancer. Apoptosis.

[14] Karki R, Sundaram B, Sharma BR, Lee S, Malireddi RS, Nguyen LN (2021). ADAR1 restricts ZBP1-mediated immune response and PANoptosis to promote tumorigenesis. Cell Rep.

[15] Pan H, Pan J, Li P, Gao J (2022). Characterization of PANoptosis patterns predicts survival and immunotherapy response in gastric cancer. Clin Immunol.

[16] Karki R, Sharma BR, Banoth B, Malireddi RS, Samir P, Tuladhar S (2020). Interferon regulatory factor 1 regulates PANoptosis to prevent colorectal cancer. JCI Insight.

[17] Gao J, Xiong A, Liu J, Li X, Wang J, Zhang L (2024). PANoptosis: bridging apoptosis, pyroptosis, and necroptosis in cancer progression and treatment. Cancer Gene Ther.

[18] Malireddi R, Karki R, Sundaram B, Kancharana B, Lee S, Samir P (2021). Inflammatory cell death, PANoptosis, mediated by cytokines in diverse cancer lineages inhibits tumor growth. ImmunoHorizons.

[19] Manohar SM (2024). At the crossroads of TNF-α signaling and cancer. Curr Mol Pharmacol.

[20] Jiang M, Qi L, Li L, Wu Y, Song D, Li Y (2021). Caspase-8: a key protein of cross-talk signal way in “PANoptosis” in cancer. Int J Cancer.

[21] Ross-Adams H, Lamb AD, Dunning MJ, Halim S, Lindberg J, Massie CM (2015). Integration of copy number and transcriptomics provides risk stratification in prostate cancer: a discovery and validation cohort study. EBioMedicine.

[22] Whitington T, Gao P, Song W, Ross-Adams H, Lamb AD, Yang Y (2016). Gene regulatory mechanisms underpinning prostate cancer susceptibility. Nat Genet.

[23] Wang Y, Kanneganti TD (2021). From pyroptosis, apoptosis and necroptosis to PANoptosis: A mechanistic compendium of programmed cell death pathways. Comput Struct Biotechnol J.

[24] Zheng Z, Li K, Yang Z, Wang X, Shen C, Zhang Y (2024). Transcriptomic analysis reveals molecular characterization and immune landscape of PANoptosis-related genes in atherosclerosis. Inflamm Res.

[25] Szklarczyk D, Gable AL, Lyon D, Junge A, Wyder S, Huerta-Cepas J (2019). STRING v11: protein–protein association networks with increased coverage, supporting functional discovery in genome-wide experimental datasets. Nucl Acids Res.

[26] Wilkerson MD, Hayes DN (2010). ConsensusClusterPlus: a class discovery tool with confidence assessments and item tracking. Bioinformatics.

[27] Love MI, Huber W, Anders S (2014). Moderated estimation of fold change and dispersion for RNA-seq data with DESeq2. Genome Biol.

[28] Hayles J (2019). Gene ontology consortium. the gene ontology resource: 20 years and still GOing strong. Nucl Acids Res.

[29] Kanehisa M, Goto S, Furumichi M, Tanabe M, Hirakawa M (2010). KEGG for representation and analysis of molecular networks involving diseases and drugs. Nucl Acids Res.

[30] Yu G, Wang LG, Han Y, He QY (2012). clusterProfiler: an R package for comparing biological themes among gene clusters. OMICS.

[31] Subramanian A, Tamayo P, Mootha VK, Mukherjee S, Ebert BL, Gillette MA (2005). Gene set enrichment analysis: a knowledge-based approach for interpreting genome-wide expression profiles. Proc Nat Acad Sci.

[32] Newman AM, Steen CB, Liu CL, Gentles AJ, Chaudhuri AA, Scherer F (2019). Determining cell type abundance and expression from bulk tissues with digital cytometry. Nat Biotechnol.

[33] Aran D, Sirota M, Butte AJ (2015). Systematic pan-cancer analysis of tumour purity. Nat Commun.

[34] Jiang P, Gu S, Pan D, Fu J, Sahu A, Hu X (2018). Signatures of T cell dysfunction and exclusion predict cancer immunotherapy response. Nat Med.

[35] Mayakonda A, Lin DC, Assenov Y, Plass C, Koeffler HP (2018). Maftools: efficient and comprehensive analysis of somatic variants in cancer. Genome Res.

[36] Engebretsen S, Bohlin J (2019). Statistical predictions with glmnet. Clin Epigenet.

[37] Tourinho-Barbosa R, Srougi V, Nunes-Silva I, Baghdadi M, Rembeyo G, Eiffel SS (2018). Biochemical recurrence after radical prostatectomy: what does it mean?. Int Braz J Urol.

[38] Shore ND, Moul JW, Pienta KJ, Czernin J, King MT, Freedland SJ (2024). Biochemical recurrence in patients with prostate cancer after primary definitive therapy: treatment based on risk stratification. Prostate Cancer Prostatic Dis.

[39] Boyle P, Ebot EM, Wilson KM, Mucci LA (2003). The epidemiology of prostate cancer. Urol Clin North Am.

[40] Narayan V, Ross AE, Parikh RB, Nohria A, Morgans AK (2021). How to treat prostate cancer with androgen deprivation and minimize cardiovascular risk. JACC CardioOncol.

[41] Hong WX, Haebe S, Lee AS, Westphalen CB, Norton JA, Jiang W (2020). Intratumoral immunotherapy for early-stage solid tumors. Clin Cancer Res.

[42] Szeto GL, Finley SD (2019). Integrative approaches to cancer immunotherapy. Trends Cancer.

[43] Lefler DS, Manobianco SA, Bashir B (2024). Immunotherapy resistance in solid tumors: mechanisms and potential solutions. Cancer Biol Ther.

[44] Cha JH, Chan LC, Song MS, Hung MC (2020). New approaches on cancer immunotherapy. Cold Spring Harb Perspect Med.

[45] Wang I, Song L, Wang BY, Kalebasty AR, Uchio E, Zi X (2022). Prostate cancer immunotherapy: recent advancements with novel treatment methods and efficacy. Am J Clin Exp Urol.

[46] Mitsogiannis I, Tzelves L, Dellis A, Issa H, Papatsoris A, Moussa M (2022). Prostate cancer immunotherapy. Expert Opin Biol Ther.

[47] Venturini NJ, Drake CG (2019). Immunotherapy for prostate cancer. Cold Spring Harb Perspect Med.

[48] Zhu M, Liu D, Liu G, Zhang M, Pan F (2023). Caspase-linked programmed cell death in prostate cancer: from apoptosis, necroptosis, and pyroptosis to PANoptosis. Biomolecules.

[49] Kulik G (2015). Personalized prostate cancer therapy based on systems analysis of the apoptosis regulatory network. Asian J Androl.

[50] Hao Y, Xie F, He J, Gu C, Zhao Y, Luo W (2024). PLA inhibits TNF-α-induced PANoptosis of prostate cancer cells through metabolic reprogramming. Int J Biochem Cell Biol.

[51] Yi X, Li J, Zheng X, Xu H, Liao D, Zhang T (2023). Construction of PANoptosis signature: novel target discovery for prostate cancer immunotherapy. Mol Ther Nucl Acids.

[52] Wang Y, Zhou J, Zhang N, Zhu Y, Zhong Y, Wang Z (2023). A novel defined PANoptosis-related miRNA Signature for predicting the prognosis and immune characteristics in clear cell renal cell carcinoma: a miRNA signature for the prognosis of ccRCC. Int J Mol Sci.

[53] Xiao Y, Yu D (2021). Tumor microenvironment as a therapeutic target in cancer. Pharmacol Ther.

[54] Shi X, Cheng X, Jiang A, Shi W, Zhu L, Mou W (2024). Immune checkpoints in B cells: unlocking new potentials in cancer treatment. Adv Sci.

[55] Peng S, Lin A, Jiang A, Zhang C, Zhang J, Cheng Q (2024). CTLs heterogeneity and plasticity: implications for cancer immunotherapy. Mol Cancer.

[56] Liu L, Xie Y, Yang H, Lin A, Dong M, Wang H (2023). HPVTIMER: a shiny web application for tumor immune estimation in human papillomavirus-associated cancers. iMeta.

[57] Kainulainen K, Niskanen EA, Kinnunen J, Mäki-Mantila K, Hartikainen K, Paakinaho V (2024). Secreted factors from M1 macrophages drive prostate cancer stem cell plasticity by upregulating NANOG, SOX2, and CD44 through NF?B-signaling. OncoImmunology.

[58] Gu Q, Qi A, Wang N, Zhou Z, Zhou X (2024). Macrophage dynamics in prostate cancer. Biomed Pharmacother.

[59] Sierra-Filardi E, Nieto C, Domínguez-Soto Á, Barroso R, Sánchez-Mateos P, Puig-Kroger A (2014). CCL2 shapes macrophage polarization by GM-CSF and M-CSF: identification of CCL2/CCR2-dependent gene expression profile. J Immunol.

[60] Caescu CI, Guo X, Tesfa L, Bhagat TD, Verma A, Zheng D (2015). Colony stimulating factor-1 receptor signaling networks inhibit mouse macrophage inflammatory responses by induction of microRNA-21. Blood.

[61] Yi-Hong C, Jing LI, Lu HO (2019). MicroRNA-155 induces macrophage polarization to M1 in toxoplasma gon-dii infection. Zhongguo Xue Xi Chong Bing Fang Zhi Za Zhi.

[62] Fei Y, Wang Z, Huang M, Wu X, Hu F, Zhu J (2023). MiR-155 regulates M2 polarization of hepatitis B virus-infected tumour-associated macrophages which in turn regulates the malignant progression of hepatocellular carcinoma. J Viral Hepat.

[63] Nicholson DW (1999). Caspase structure, proteolytic substrates, and function during apoptotic cell death. Cell Death Differ.

[64] Lamkanfi M, Kanneganti TD (2010). Caspase-7: a protease involved in apoptosis and inflammation. Int J Biochem Cell Biol.

[65] Kim SH, Baek KH (2022). Ovarian tumor deubiquitinase 6A regulates cell proliferation via deubiquitination of nucleolin and caspase-7. Int J Oncol.

[66] Nishikura K (2010). Functions and regulation of RNA editing by ADAR deaminases. Annu Rev Biochem.

[67] Ramírez-Moya J, Miliotis C, Baker AR, Gregory RI, Slack FJ, Santisteban P (2021). An ADAR1-dependent RNA editing event in cyclin-dependent kinase CDK13 promotes thyroid cancer hallmarks. Mol Cancer.

[68] Yu H, Bai K, Cheng Y, Lv J, Song Q, Yang H (2023). Clinical significance, tumor immune landscape and immunotherapy responses of ADAR in pan-cancer and its association with proliferation and metastasis of bladder cancer. Aging.

[69] Qian W, Wang J, Van Houten B (2013). The role of dynamin-related protein 1 in cancer growth. Expert Opin Ther Targets.

[70] Inoue-Yamauchi A, Oda H (2012). Depletion of mitochondrial fission factor DRP1 causes increased apoptosis in human colon cancer cells. Biochem Biophys Res Commun.

[71] Xie Q, Wu Q, Horbinski CM, Flavahan WA, Yang K, Zhou W (2015). Mitochondrial control by DRP1 in brain tumor initiating cells. Nat Neurosci.

[72] Liston P, Roy N, Tamai K, Lefebvre C, Baird S, Cherton-Horvat G (1996). Suppression of apoptosis in mammalian cells by NAIP and a related family of IAP genes. Nature.

[73] Martinon F, Tschopp J (2007). Inflammatory caspases and inflammasomes: master switches of inflammation. Cell Death Differ.

[74] Chen YK, Huse SS, Lin LM (2011). Expression of inhibitor of apoptosis family proteins in human oral squamous cell carcinogenesis. Head Neck.

[75] Choi J, Hwang YK, Choi YJ, Yoo KE, Kim JH, Nam SJ (2007). Neuronal apoptosis inhibitory protein is overexpressed in patients with unfavorable prognostic factors in breast cancer. J Korean Med Sci.

